# Supplementary material for: The effect of intrapartum prolonged oxygen exposure on fetal metabolic status: secondary analysis from a randomized controlled trial
Source: Front Endocrinol (Lausanne). 2023 Jun 27;14:1204956. doi: 10.3389/fendo.2023.1204956 (PMC10335765; doi:10.3389/fendo.2023.1204956)
Supplement: Supplementary file 3 [file DataSheet_3.zip › Result-X101SC21092977-Z01-J001-B1-42/3.MetDiffScreening/O.vs.A/O.vs.A_neg.xls.volcano.pdf]

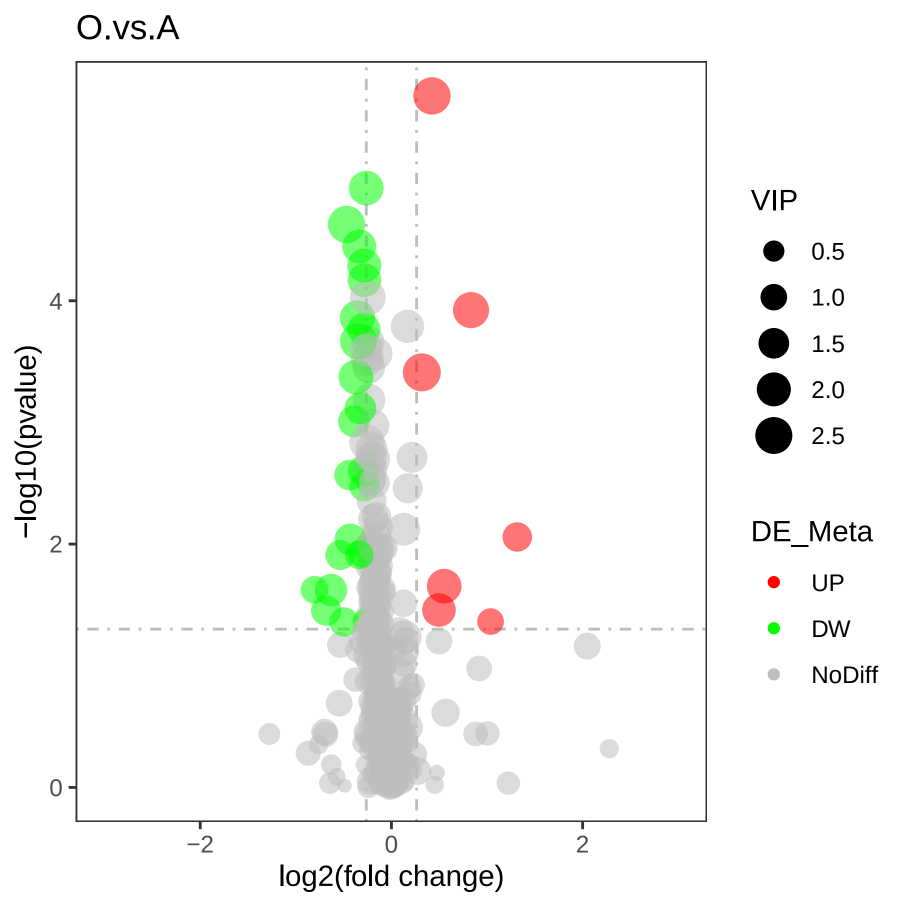

O.vs.A
-log10(pvalue)
log2(fold change)
4
2
0
−2
0
2
VIP
0.5
1.0
1.5
2.0
2.5
DE_Meta
UP
DW
NoDiff
